# Echocardiographic Parameters and the Risk of Incident Atrial Fibrillation: The Suita Study

**DOI:** 10.2188/jea.JE20180251

**Published:** 2020-04-05

**Authors:** Aya Higashiyama, Yoshihiro Kokubo, Makoto Watanabe, Yoko Masukata Nakao, Tomonori Okamura, Akira Okayama, Yoshihiro Miyamoto

**Affiliations:** 1 Department of Preventive Cardiology, National Cerebral and Cardiovascular Center, Osaka, Japan; 2 Department of Preventive Medicine and Public Health, Keio University, Tokyo, Japan; 3 Research Institute of Strategy for Prevention, Tokyo, Japan

**Keywords:** left atrial dimension, echocardiography, atrial fibrillation

## Abstract

**Background:**

Left atrial dimension (LAD) and other parameters of echocardiography have been reported to be associated with the risk of atrial fibrillation (AF). However, few studies have investigated the associations between echocardiographic parameters and the risk of AF in the Asian general population, which has a low AF incidence.

**Methods:**

A prospective cohort study was performed in 1,424 individuals in the Suita study with echocardiographic parameters, including LAD, and no history of AF. After echocardiography, the participants were followed using 12-lead electrocardiography and questionnaires to detect AF incidence. The multivariable-adjusted hazard ratios (HRs) of echocardiographic parameters for AF incidence were estimated after adjustment for the risk factors of the AF risk score.

**Results:**

During the median 6.0 years of follow-up, 31 AF cases occurred. The multivariable-adjusted HR of a 1-mm increase in LAD for AF was 1.18 (95% confidence interval [CI], 1.08–1.28). The multivariable-adjusted HR for AF of a 1-standard-deviation increase in LAD was higher than that of left ventricular internal dimensions in diastole, left ventricular mass, ejection fraction, and percent fractional shortening, and it was the only significant factor. In 667 participants with both LAD and LA volume (LAV) measurements, LAD and LAV were independently associated with the risk of AF incidence.

**Conclusions:**

LAD on echocardiography was an independent risk factor of incident AF in the Japanese population. LAD might be useful for identifying individuals with a high risk of AF in health check-ups of the general population.

## INTRODUCTION

Atrial fibrillation (AF) is associated with increased risks of stroke, myocardial infarction, heart failure, dementia, chronic kidney disease, and mortality.^1^^,^^2^ Although the prevalence of AF in Japan has been lower than that in Western countries,^3^ it is estimated to increase in 2050 based on population projections.^4^ Thus, prevention of AF is an important challenge to extend healthy life expectancy in the Japanese population.

In the strategy for AF prevention, not only controlling AF risk factors, but also effectively and non-invasively finding individuals at risk is important. Transthoracic echocardiography is a non-invasive tool to examine cardiac function and morphology. Previous studies in the Western population have reported that left atrial dimension (LAD) and other echocardiographic parameters are associated with the risk of incident AF.^5^^,^^6^ However, to the best of our knowledge, no previous study has investigated the associations between echocardiographic parameters and AF incidence with adjustment for AF risk factors in the Asian general population, which has a low AF incidence.

To investigate the associations between echocardiographic parameters and AF incidence in the Japanese general population, a prospective cohort study involving the participants of the Suita study was performed.

## METHODS

### Study participants

The Suita study,^7^ a cohort study of cardiovascular disease (CVD), was established in Suita City, Osaka, Japan. In the study, 6,485 participants, who were randomly selected from the municipal population registry, participated in a baseline survey at the National Cardiovascular Center (NCVC) between September 1989 and February 1994, and they have been followed with biennial examinations. Since 2007, echocardiography was performed once in the participants.

From July 1, 2007 to September 30, 2017, echocardiography was performed in 2,175 participants with no AF history and no symptoms of heart failure based on questionnaires. Among them, individuals with mitral stenosis on echocardiography (*n* = 4), technically inadequate or unavailable LAD data (*n* = 43), missing data for the covariates (*n* = 6), fasting <8 hours (*n* = 56), and those lost to follow-up (*n* = 255) were excluded. From the remaining 1,811 participants with LAD measurements, 387 individuals were additionally excluded due to technically inadequate or unavailable data for left ventricular internal dimensions in diastole and systole (LVIDd and LVIDs), interventricular septum (IVS), left ventricular posterior wall (LVPW), and ejection fraction (EF). The remaining 1,424 participants (590 men and 834 women) were then analyzed in the present study (eFigure 1). Left atrial volume (LAV) was available in only 667 participants (235 men and 432 women), because LAV measurement was started in 2010.

Written informed consent was given by all participants. The present study was approved by the Institutional Review Board of the NCVC (M19-005-7).

### Echocardiography

A well-trained technician performed the echocardiography (EUB-6500 and 2.5-MHz transducer [EUP-S50A], Hitachi, Tokyo, Japan). LAD was determined according to the American Society of Echocardiography recommendations in 2005 using a trailing-edge-to-leading-edge convention to measure the maximal distance between the posterior aortic root wall and the posterior left atrial wall at end systole.^8^ Parasternal long axis left ventricular wall thicknesses (IVS and LVPW) and LVID were measured using M-mode at diastole and systole, and LVM was calculated using the Cube formula^9^: LVM(g) = 0.8 * 1.04{(LVIDd + IVS + LVPW)^3^ − LVIDd^3^} + 0.6. EF and LAV were measured using the modified Simpson method. LVM and LAV were standardized for body size by dividing LVM and LAV by body surface area calculated using Fujimoto’s method.^10^ Percent fractional shortening (%FS) was calculated as {(LVIDd − LVIDs)/LVIDd} * 100.

### Other measurements

The following measurements were performed on the day of echocardiography. Well-trained nurses obtained information on smoking,^11^ alcohol consumption, and medical histories. The assessment of alcohol consumption was previously reported.^12^ Briefly, for current drinkers, the frequency of alcohol consumption during a typical week and the total alcohol intake on each occasion were asked, and the average alcohol intake per day was calculated. The usual daily intake of alcohol was assessed in units of “gou” (corresponding to 23 g of ethanol), and half a gou was defined as one drink, a value nearly equal to a ‘standard drink’ in other countries.^13^ Excessive drinking was defined in the present study as current alcohol intake ≥2.0 drinks/day.^14^

Well-trained nurses measured participants’ blood pressure (BP) in the right arm twice with the participant in a seated position after 5 minutes of rest using a standard mercury sphygmomanometer. The average of the measurements was used in the analyses. Height in socks and weight in light clothing were measured. The body mass index (BMI) was calculated as weight (kg) divided by the square of height (m^2^). Blood samples were collected at the NCVC after the participants had fasted for at least 8 hours. The samples were centrifuged immediately, and a routine blood examination that included serum total cholesterol (TC), high-density lipoprotein cholesterol (HDL-C), and triglyceride (TG) levels was then carried out. Non-HDL cholesterol (non-HDL-C) was calculated by subtracting HDL-C from TC.

Standard 12-lead electrocardiograms (ECGs) were obtained from all participants while in the supine position. Each record was coded independently using the Minnesota Code by two well-trained physicians.

### Follow-up and endpoint determination

After the echocardiography, participants were followed for AF incidence. They were invited to the routine Suita health check-up examination, including ECGs. Well-trained nurses obtained the medical history questionnaires. The participants were diagnosed as having AF when AF (Minnesota Codes 8-3-1 and 8-3-3) was observed on an ECG or the research doctors confirmed that the participants had a history of AF after echocardiography through careful consideration of the participants’ medical history.

The endpoint of each follow-up was whichever of the following occurred first: (1) the date of the first AF event; or (2) the date of the last health examination (up to March 31, 2018).

### Statistical analysis

To show the baseline characteristics of the participants, continuous variables are expressed as means (standard deviations [SDs]), medians, or range, and categorical data are expressed as numbers or percentages. Age-adjustment of continuous variables was performed using ANCOVA, and that of categorical variables was performed via a direct method using Suita city population statistics in 2008 as the standard population.

Cox proportional hazards models were used to evaluate the associations between echocardiographic parameters and AF incidence. The echocardiographic parameters were LAD, the LAD tertiles, and 1-SD increases of LAD, LVIDd, LVM, LVMI, EF, and %FS. Age- and sex-adjusted and multivariable-adjusted hazard ratios (HRs) were estimated in the models. The covariates included in the model were age, sex, overweight (BMI ≥25.0 kg/m^2^), hypertension (systolic BP ≥140 mm Hg and/or diastolic BP ≥90 mm Hg and/or present medication for hypertension), excessive alcohol drinking, current smoking, non-HDL-C, past history of coronary heart disease (CHD), the presence of valvular heart disease on echocardiography (aortic stenosis [AS], and mitral regurgitation [MR] and/or aortic regurgitation [AR] moderate or greater), and the presence of arrhythmia other than AF. These covariates were determined by reference to the risk factors of the AF risk score in the Suita study.^15^ In 681 participants, the risks for AF of LAD, LAV, LAVI, and 1-SD increases of these three parameters were also estimated.

All statistical analyses were performed using SPSS statistical software, version 22.0 J (IBM, Tokyo, Japan). *P* < 0.05 (two-tailed) was considered significant.

## RESULTS

During 8,118 person-years of follow-up, 31 incident AF cases were observed. The sex-specific follow-up data are presented in Table 1.

**Table 1.** Number of AF cases, median follow-up period and crude incidence in 1,424 participants: the Suita study

	Men	Women
Number of participants	590	834
Number of cases of incident AF	22	9
Median follow-up and range, years	6.0 (1.72–10.43)	6.0 (1.74–10.43)
Total follow-up, person-years	3,508	4,610
Crude incidence, /1,000 person-years	6.3	2.0

AF, atrial fibrillation.

The AF risk factors at baseline are presented according to LAD tertiles in Table 2. Age, BMI, BP, glucose, and the percentage of male participants, overweight, medication for hypertension and diabetes, smoking, and excessive drinking were higher in higher LAD tertiles.

**Table 2.** LAD tertiles and AF risk factors of 1,424 participants: the Suita study

	LAD 1st tertile (<29.8 mm)	LAD 2nd tertile (29.8–33.7 mm)	LAD 3rd tertile (≥33.8 mm)
Number of participants	475	483	466
Men, %	27.2	42.4	54.9
Age, years	66	66	69
BMI, kg/m^2 a^	20.7	22.5	24.3
Overweight, %^b^	4.1	18.6	51.0
Systolic blood pressure, mm Hg^a^	123	128	134
Diastolic blood pressure, mm Hg^a^	75	78	81
Medication for hypertension, %^b^	11.9	18.1	32.9
Hypertension, %^b^	21.7	36.1	51.5
Glucose, mg/dL^a^	99	102	105
HbA1c, %^a^	5.4	5.4	5.5
Medication for diabetes, %^b^	2.6	5.2	7.6
Non-HDL cholesterol, mg/dL^a^	149	150	149
Medication for dyslipidemia, %^b^	10.8	18.9	18.1
Arrhythmia other than atrial fibrillation, %^b^	9.0	15.1	8.4
Valvular heart disease, %^b^	1.9	2.0	1.6
History of coronary heart disease, %^b^	1.8	1.4	2.5
Current smoking, %^b^	7.2	14.3	26.1
Current excessive alcohol drinking, %^b^	10.5	14.4	28.1

AF, atrial fibrillation; BMI, body mass index; Hb, hemoglobin; HDL, high density lipoprotein; LAD, left atrial dimension.

Overweight is defined as BMI ≥25.0 kg/m^2^.

Valvular heart disease includes history of valvular heart disease, aortic regurgitation (moderate or greater), mitral regurgitation (moderate or greater), and/or aortic stenosis (AS) in echocardiography.

Excessive alcohol drinking is defined as drinking 2 drinks and more/day.

^a^Age adjusted using analysis of covariance (ANCOVA).

^b^Age adjusted using direct method with 5-year age groups population of Suita city in 2008.

Table 3 presents the age and sex-adjusted and the multivariable-adjusted HRs of LAD and the LAD tertiles for incident AF. The multivariable-adjusted HR of a 1-mm increase in LAD for AF was 1.18 (95% confidence interval [CI], 1.08–1.28), and that of the highest LAD tertile for AF compared to that of the lowest was 4.61 (95% CI, 1.30–16.30).

**Table 3.** Risk of LAD increase for the incidence of AF in 1,424 participants: the Suita study

	*n*	Cases	Range of the parameter mean (SD)	Age and sex-adjusted HR and 95% CI	Multivariable-adjusted HR and 95% CI
Left atrial dimension	1,424	31	15.1–47.4	1.20 (1.11–1.30)	1.18 (1.08–1.28)
			31.8 (4.7)		
Tertile of left atrial dimension					
T1 (<29.8 mm)	475	3		1.00	1.00
T2 (29.8–33.7 mm)	483	7		1.78 (0.46–6.93)	1.80 (0.46–7.06)
T3 (≥33.8 mm)	466	21		5.41 (1.59–18.44)	4.61 (1.30–16.30)
Total	1,424	31			
Trend *P*				<0.01	<0.01

AF, atrial fibrillation; BMI, body mass index; CI, confidence interval; SD, standard deviation; HDL-C, high density lipoprotein-cholesterol; HR, hazard ratio; LAD, left atrial dimension.

Multivariable-adjusted HR: age, sex, overweight (BMI ≥25.0 kg/m^2^), hypertension (systolic blood pressure ≥140 mm Hg and/or diastolic blood pressure ≥90 mm Hg and/or present medication for hypertension), current excessive alcohol drinking, current smoking, non-HDL-C, past history of coronary heart disease, valvular heart disease, and the presence of arrhythmia other than AF.

Table 4 presents the age- and sex-adjusted and the multivariable-adjusted HRs of a 1-SD increase of the echocardiographic parameters, including LAD, for incident AF. Among the parameters, a 1-SD increase of LAD showed the highest and the only significant increase of HR.

**Table 4.** Risk of 1-SD-increase of LAD and other parameters for the incidence of AF in 1,424 participants: the Suita study

	*n*	Cases	Range of the parameter mean (SD)	Parameter increment	Age and sex-adjusted HR and 95% CI	Multivariable-adjusted HR and 95% CI
Left atrial dimension, mm	1,424	31	15.1–47.4	4.7	2.35 (1.61–3.42)	2.16 (1.45–3.24)
			31.8 (4.7)			
Left ventricular internal dimension, mm	1,424	31	30.5–60.7	4.5	1.26 (0.85–1.87)	1.14 (0.76–1.72)
			45.5 (4.5)			
Left ventricular mass, g	1,424	31	33.8–255.4	33.3	1.60 (1.12–2.29)	1.36 (0.91–2.04)
			123.2 (33.3)			
Left ventricular mass index, g/m^2^	1,424	31	21.1–159.3	17.4	1.45 (1.06–1.99)	1.30 (0.92–1.86)
			80.0 (17.4)			
Ejection fraction, %	1,424	31	42.3–88.6	6.5	1.06 (0.75–1.50)	1.05 (0.75–1.48)
			68.2 (6.5)			
Percent FS, %	1,424	31	10.6–71.1	6.0	0.91 (0.63–1.32)	0.92 (0.64–1.32)
			38.4 (6.0)			

AF, atrial fibrillation; BMI, body mass index; CI, confidence interval; FS, fractional shortening; HDL-C, high density lipoprotein-cholesterol; HR, hazard ratio; LAD, left atrial dimension; SD, standard deviation.

Multivariable-adjusted HR: age, sex, overweight (BMI ≥25.0 kg/m^2^), hypertension (systolic blood pressure ≥140 mm Hg and/or diastolic blood pressure ≥90 mm Hg and/or present medication for hypertension), current excessive alcohol drinking, current smoking, non-HDL-C, past history of coronary heart disease, valvular heart disease, and the presence of arrhythmia other than AF.

The HRs of LAD, LAV, and LAVI, and those of 1-SD increase of the parameters for incident AF in 681 participants are presented in eTable 1 and eTable 2. All three parameters were associated with a significantly increased risk of incident AF.

In 1,416 participants (30 AF events) with early diastolic filling velocity/atrial filling velocity (E/A) data available, the HR of those with E/A <0.7 was 1.27 (95% CI, 0.46–3.51), and that of those with E/A >1.5 was 5.36 (95% CI, 1.66–17.28), compared to those with E/A ≥0.7 and E/A ≤1.5, respectively (data not shown).

## DISCUSSION

In the present study, LAD measured using echocardiography was associated with the risk of incident AF in a relatively healthy Japanese population, after adjustment for the risk factors in the AF risk score of the Suita study. Furthermore, a 1-SD increase of LAD showed the highest HR for incident AF among the examined continuous echocardiographic parameters of cardiac structure and function.

To the best of our knowledge, the present study is the first population-based cohort study to show the risk of LAD enlargement measured using echocardiography for incident AF in an Asian general population, which has a low AF incidence compared to Western nations. The Framingham Heart Study reported that LAD, the sum of wall thicknesses, and %FS were independent predictors of incident AF in whites.^5^ In the present study, LVM via the Cube formula and EF via the modified Simpson method were used, and they are now often measured. The ARIC study reported that only addition of LAD to the CHARGE AF score resulted in a significant improvement in the C-statistic for prediction of AF incidence in blacks, among the ECG parameters, such as LVID, %FS, LAD, LVMI, E/A (<0.7 and >1.5) and EF (<50%).^6^ Although the HR of the participants with E/A >1.5 was high in the present study, the results seem to be consistent with those in the ARIC study. In a cross-sectional study in Korea, LVIDd, LVIDs, and EF were associated with the prevalence of AF.^16^ However, the risk of the parameters for incident AF has not been investigated.

The incidence of AF was 4.3/1,000 person-years in men and women aged 60–79 years. In our previous Suita study, we developed a 10-year AF risk score, and the AF incidence was 6.7/1,000 person-years among those aged 60–79 years.^15^ The lower incidence in the present study might be partly explained by the fact that the data of individuals who died from the disease caused by AF after echocardiography, and those lost to follow-up, were not included. Future study is needed to investigate the risk of LAD enlargement for AF incidence in all individuals with echocardiographic data after the latest determination of the AF-related cause of death in the Suita study. Thus, the incidence in our previous study should be considered the accurate AF incidence for the Suita study, and the results of the present study should be interpreted with an understanding of the limited definition of the end of follow-up.

The recent guideline for echocardiography recommends that the anteroposterior linear dimension should not be used as the sole measure of LA size, because LAD may not represent an accurate picture of LA size.^9^ However, in the present study, both increased LAD and LAV were independent risk factors for AF. To prevent AF in the general population, early detection of individuals at risk is important. Compared to LAV, LAD is more basic and easier to measure on echocardiography. With the recent development of portable echocardiography machines, screening of LAD in the general population could be useful in health check-ups to identify individuals at high risk of AF.

In previous studies that investigated the association between LV systolic function and AF incidence in the general population, %FS was often used.^5^^,^^6^ In the Framingham Heart Study and the ARIC study, lower %FS was associated with a higher risk of AF,^5^^,^^6^ with the lowest risk among the participants with a %FS of 32–36% in the ARIC study.^6^ The results in the present study were similar to those in the previous studies, but the risk decrease with higher %FS was not evident due to the relatively healthy participants with a low AF incidence and higher %FS in the present study.^5^^,^^6^ Furthermore, in the ARIC study, EF <50% was associated with an increased risk of AF.^6^ However, the present study could not investigate the association between EF <50% and incident AF due to the small number of participants with EF <50% (*n* = 6). The results of decreased or increased E/A compared to normal E/A in the present study were similar to those in the ARIC study.

Possible mechanisms of incident AF are mechanical overload of the LA, autonomic modulation, and biochemical electrical remodeling in the atrium.^17^^–^^19^ and these factors are combined simultaneously and sequentially.^3^ Of these factors, mechanical overload is common as the underlying condition of AF.^3^ Stretching of the atrial appendage leads to remodeling of the anatomy and physiology of the LA and increases dispersion of atrial refractoriness.^5^^,^^20^^,^^21^ Impairment of LV end-diastolic filling pressure and LA emptying caused by LV size and dysfunction could also lead to the development of AF through worsening atrial structural remodeling.^6^ AF itself reduces atrial contractility, contributes to atrial dilatation and more remodeling, and leads to the progression and perpetuation of the nature of AF.^22^ In addition, hypertension, CHD, age, and inflammation act as triggers and propagating factors that directly affect the measures of cardiac structure and function and increase AF risk.^6^

The limitations of the present study are as follows. First, AF incidence was small. Because the incidence in the present study was limited to persistent and/or paroxysmal AF detected using 12-lead ECG and self-reported history in the follow-up health check-ups, there could have been missed AF cases. Second, the participants could have been limited to a relatively healthy population among the participants in the Suita study, because the participants in the present study were those who could repeatedly visit for the follow-up health check-ups. Thus, interpretation of the results in the present study should be carefully considered in the clinical situation. Finally, LAVI was measured only in about half of the participants in the present study.

In conclusion, LAD enlargement on echocardiography was an independent risk factor for incident AF in a relatively healthy Japanese general population, and a 1-SD increase of LAD showed the highest HR for AF among the examined continuous echocardiographic parameters of cardiac structure and function. With the recent development of portable echocardiography machines, LAD could be useful to detect future risk for AF in the general population in community health check-ups.

## APPENDIX A. SUPPLEMENTARY DATA

eFigure 1. Inclusion and exclusion criteria of participants: the Suita study in 2007–2018. AF: atrial fibrillation, LAD: left atrial dimension, LVID: left ventricular internal dimension, IVS: interventricular septum, LVPW: left ventricular posterior wall, EF: ejection fraction, LAV: left atrial volume.

eTable 1. Risks of LAD, LAV and LAVI increases for the incidence of AF in 667 participants: the Suita study

eTable 2. Risks of 1-SD increase of LAD, LAV, and LAVI for the incidence of AF in 667 participants: the Suita study

## References

[1] Benjamin EJ, Wolf PA, D’Agostino RB, Silbershatz H, Kannel WB, Levy D Impact of atrial fibrillation on the risk of death: the Framingham Heart Study. Circulation. 1998;98:946–952. 10.1161/01.CIR.98.10.946 9737513

[2] Ohsawa M, Okamura T, Tanno K, Risk of stroke and heart failure attributable to atrial fibrillation in middle-aged and elderly people: results from a five-year prospective cohort study of Japanese community dwellers. J Epidemiol. 2017;27:360–367. 10.1016/j.je.2016.08.012 28390793 PMC5549250

[3] JCS Joint Working Group Guidelines for Pharmacotherapy of Atrial Fibrillation (JCS 2013). Circ J. 2014;78:1997–2021. 10.1253/circj.CJ-66-0092 24965079

[4] Inoue H, Fujiki A, Origasa H, Prevalence of atrial fibrillation in the general population of Japan: an analysis based on periodic health examination. Int J Cardiol. 2009;137:102–107. 10.1016/j.ijcard.2008.06.029 18691774

[5] Vaziri SM, Larson MG, Benjamin EJ, Levy D Echocardiographic predictors of nonrheumatic atrial fibrillation. The Framingham Heart Study. Circulation. 1994;89:724–730. 10.1161/01.CIR.89.2.724 8313561

[6] Bekwelem W, Misialek JR, Konety S, Echocardiographic measures of cardiac structure and function are associated with risk of atrial fibrillation in blacks: the Atherosclerosis Risk in Communities (ARIC) study. PLoS One. 2014;9:e110111. 10.1371/journal.pone.0110111 25330035 PMC4199625

[7] Okamura T, Kokubo Y, Watanabe M, Low-density-lipoprotein cholesterol and non-high density lipoprotein cholesterol and the incidence of cardiovascular disease in an urban Japanese cohort study: the Suita study. Atherosclerosis. 2009;203:587–592. 10.1016/j.atherosclerosis.2008.07.020 18783774

[8] Lang RM, Bierig M, Devereux RB, ; Chamber Quantification Writing Group; American Society of Echocardiography’s Guidelines and Standards Committee; European Association of Echocardiography Recommendations for chamber quantification: a report from the American Society of Echocardiography’s Guidelines and Standards Committee and the Chamber Quantification Writing Group, developed in conjunction with the European Association of Echocardiography, a branch of the European Society of Cardiography. J Am Soc Echocardiogr. 2005;18:1440–1463. 10.1016/j.echo.2005.10.005 16376782

[9] Lang RM, Badano LP, Mor-Avi V, Guidelines and standards recommendations for cardiac chamber quantification by echocardiography in adults: an update from the American Society of Echocardiography and the European Association of Cardiovascular Imaging. J Am Soc Echocardiogr. 2015;28:1–39.e14. 10.1016/j.echo.2014.10.003 25559473

[10] Fujimoto S, Watanabe T, Sakamoto A, Yukawa K, Morimoto K Studies on the physical surface area of Japanese Part 18 calculation formulas in three stages over all age. Nihon Eiseigaku Zasshi. 1968;23:443–450. 10.1265/jjh.23.443 5752712

[11] Higashiyama A, Okamura T, Ono Y, Watanabe M, Kokubo Y, Okayama A Risk of smoking and metabolic syndrome for incidence of cardiovascular disease-comparison of relative contribution in urban Japanese population: the Suita study. Circ J. 2009;73:2258–2263. 10.1253/circj.CJ-09-0264 19838005

[12] Higashiyama A, Wakabayashi I, Ono Y, Association with serum gamma-glutamyltransferase levels and alcohol consumption on stroke and coronary artery disease. The Suita study. Stroke. 2011;42:1764–1767. 10.1161/STROKEAHA.110.608307 21512179

[13] International Center for Alcohol Policies (ICAP). What is a “standard drink”? ICAP Reports No. 5 ICAP, 1998, Washington, DC.

[14] Japan Health Promotion and Fitness Foundation. Health Japan 21. Ministry of Health, Labour and Welfare. Report on Health Japan 21 Plan Study Committee and Health Japan 21 Plan Development Committee.

[15] Kokubo Y, Watanabe M, Higashiyama A, Nakao YM, Kusano K, Miyamoto Y Development of a basic risk score for incident atrial fibrillation in a Japanese general population - The Suita Study. Circ J. 2017;81:1580–1588. 10.1253/circj.CJ-17-0277 28539563

[16] Park HC, Park JK, Choi SI, Prevalence of atrial fibrillation and relation to echocardiographic parameters in a healthy asymptomatic rural Korean population. J Korean Med Sci. 2015;30:1078–1084. 10.3346/jkms.2015.30.8.1078 26240485 PMC4520938

[17] Yamashita T, Murakawa Y, Hayami N, Short-term effects of rapid pacing on mRNA level of voltage-dependent K(+) channels in rat atrium: electrical remodeling in paroxysmal atrial tachycardia. Circulation. 2000;101:2007–2014. 10.1161/01.CIR.101.16.2007 10779469

[18] Coumel P. *Neural aspects of paroxysmal atrial fibrillation. Atrial Fibrillation: Mechanisms and Management*. Raven Press; 1992:109–125.

[19] Maisel WH Autonomic modulation preceding the onset of atrial fibrillation. J Am Coll Cardiol. 2003;42:1269–1270. 10.1016/S0735-1097(03)00959-8 14522494

[20] Tsang TS, Barnes ME, Bailey KR, Left atrial volume: important risk marker of incident atrial fibrillation in 1655 older men and women. Mayo Clin Proc. 2001;76:467–475. 10.4065/76.5.467 11357793

[21] Wang TJ, Parise H, Levy D, Obesity and the risk of new-onset atrial fibrillation. JAMA. 2004;292:2471–2477. 10.1001/jama.292.20.2471 15562125

[22] Allessie M, Ausma J, Schotten U Electrical, contractile and structural remodeling during atrial fibrillation. Cardiovasc Res. 2002;54:230–246. 10.1016/S0008-6363(02)00258-4 12062329

