# Supplementary material for: Echocardiographic Parameters and the Risk of Incident Atrial Fibrillation: The Suita Study
Source: J Epidemiol. 2020 Apr 5;30(4):183–7. doi: 10.2188/jea.JE20180251 (PMC7064552; doi:10.2188/jea.JE20180251)
Supplement: Supplementary file 1 [file je-30-183-s001.pdf]

eTable 1. Risks of LAD, LAV and LAVI increases for the incidence of AF in 667 participants: the Suita study

	n	Cases	Range of the parameter mean (SD)	Age and sex-adjusted HR and 95% CI	Multivariable-adjusted HR and 95% CI
Left atrial dimension, mm	667	10	15.8–44.3<br>31.3 (4.8)	1.28 (1.10 – 1.50)	1.22 (1.04 – 1.44)
Left atrial volume, mL	667	10	11.1–93.3<br>32.9 (11.1)	1.07 (1.03 – 1.12)	1.06 (1.02 – 1.10)
Left atrial volume index, mL/m^2	667	10	7.8–71.2<br>21.6 (7.3)	1.10 (1.04 – 1.17)	1.10 (1.02 – 1.17)

AF, atrial fibrillation; BMI, body mass index; CI, confidence interval; HDL-C, high density lipoprotein-cholesterol; HR, hazard ratio; LAD, left atrial dimension; LAV, left atrial volume; LAVI, left atrial volume index; SD, standard deviation.

Multivariable-adjusted HR: age, sex, overweight (BMI ≥25.0 kg/m^2), hypertension (systolic blood pressure ≥140 mm Hg and/or diastolic blood pressure ≥90 mm Hg and/or present medication for hypertension), current excessive alcohol drinking, and non-HDL-C.

eTable 2. Risks of 1-SD increase of LAD, LAV, and LAVI for the incidence of AF in 667 participants: the Suita study

	n	Cases	Range of the parameter mean (SD)	Parameter increment	Age and sex-adjusted HR and 95% CI	Multivariable-adjusted HR and 95% CI
Left atrial dimension, mm	667	10	15.8–44.3<br>31.3 (4.8)	4.8	3.30 (1.58 – 6.89)	2.63 (1.20 – 5.80)
Left atrial volume, mL	667	10	11.1–93.3<br>32.9 (11.1)	11.1	2.17 (1.38 – 3.42)	1.94 (1.18 – 3.18)
Left atrial volume index, mL/m^2	667	10	7.8–71.2<br>21.6 (7.3)	7.3	2.00 (1.29 – 3.09)	1.91 (1.17 – 3.12)

AF, atrial fibrillation; BMI, body mass index; CI, confidence interval; HDL-C, high density lipoprotein-cholesterol; HR, hazard ratio; LAD, left atrial dimension; LAV, left atrial volume; LAVI, left atrial volume index; SD, standard deviation.

Multivariable-adjusted HR: age, sex, overweight (BMI ≥25.0 kg/m^2), hypertension (systolic blood pressure ≥140 mm Hg and/or diastolic blood pressure ≥90 mm Hg and/or present medication for hypertension), current excessive alcohol drinking, and non-HDL-C.

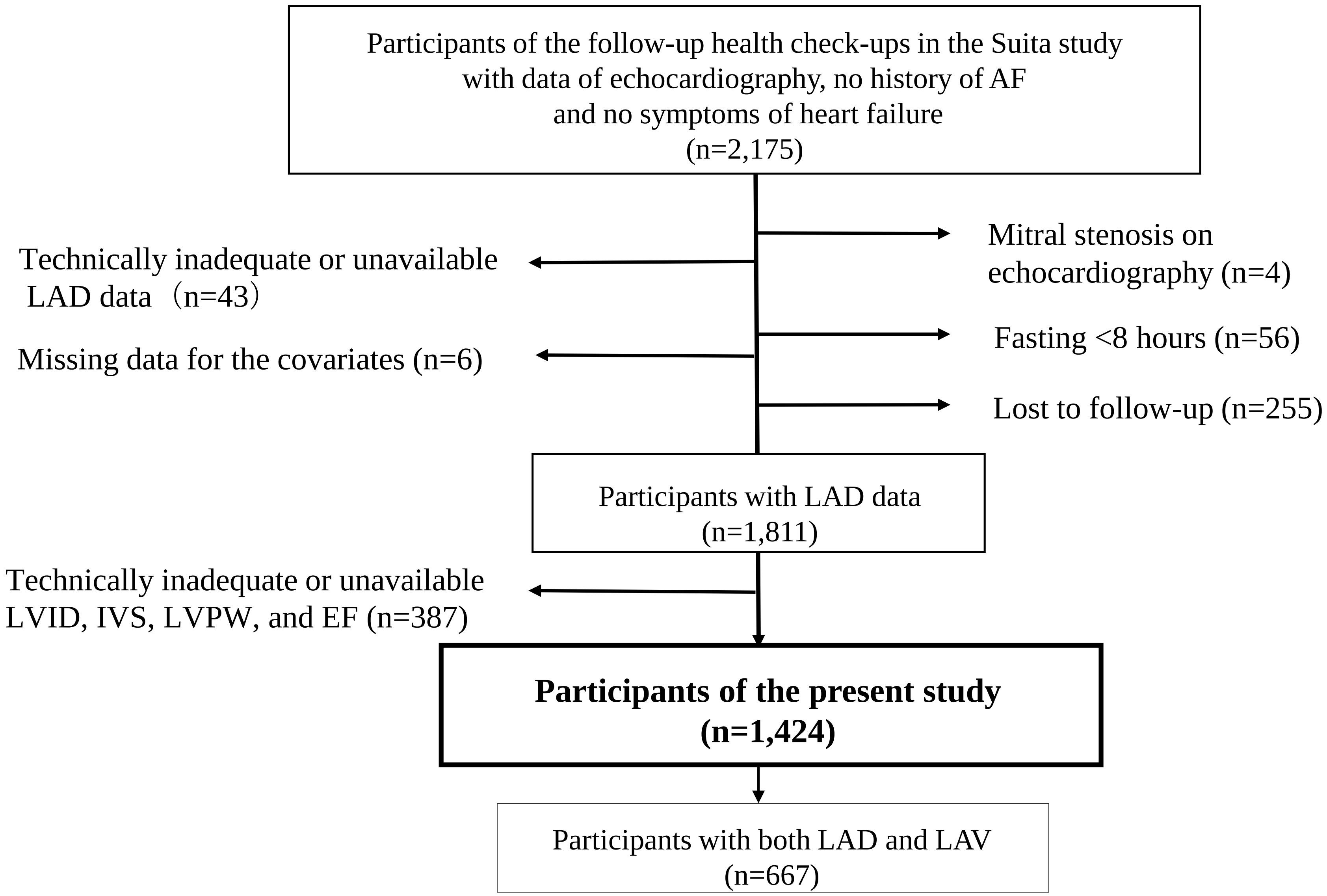

eFigure 1. Inclusion and exclusion criteria of participants: the Suita study in 2007–2018. AF: atrial fibrillation, LAD: left atrial dimension, LVID: left ventricular internal dimension, IVS: interventricular septum, LVPW: left ventricular posterior wall, EF: ejection fraction, LAV: left atrial volume.
